# Scientific News

**Published:** 1880-05

**Authors:** 


### Scientific News.

Jupiter's diameter is about eleven times that of our earth, and his mean density is about a quarter of that of the earth, or about a third more than water. Now, a bulky body may be composed of heavy materials, and still, as a whole, be light, like an iron ship or a lump of pumice-stone, that will float in water. The pumice lump is light on account of its vesicular formation, so that the mass consists of heavy felspathic material and the air it contains. Extract the air, and the pumice loses its floating power, though still far from heavy in proportion to its bulk. Most of the earth's crust is formed of solids much heavier than water. Granites are more than two and a half times heavies than water, slaty rocks much about the same, and so are ordinary limestones, the variations of all being from about 2.5 to 2.9. The ironstone group contains denser minerals; red hematite has a specific gravity of 4.5; magnetic ironstone, 4 5 to 5.2, &c., and many other ores are heavy. At some remote period, when only a part of the now solid earth had been condensed from gaseous and vapory matter, our planet might have had a mean density like that of Jupiter, as its rocky materials contain between 40 and 50 per per cent. of oxygen; and while condensations and chemical combinations were going on rapidly our globe must have been the scene of

"Thunders, lightnings, and prodigious storms."

And it is probable that certain stars which have suddenly blazed forth with passing splendor have exhibited to us the spectacle of conflagrations extending over millions and billions of square miles. Color changes in Jupiter—such as those noticed by Mr. Browning and the writer in 1869–70—may have been caused by soda-flames, though not fierce enough or extensive enough to add materially to his ordinary luminosity, which is estimated as always exceeding, though not in a very high degree, what it would be by mere reflection of light received from the sun.—*Belgravia.*

R. Rudolphi reports, in the *Gazetta Medica Italiana*, the following observation made on himself: Being seized with a severe coryza, he happened to chew one or two twigs of the *eucalyptus*, at the same time swallowing the saliva secreted, which had a bitter and aromatic flavor, to his surprise he found that, in the course of half an hour the nasal catarrh had disappeared. Some days later he was seized with another attack from a fresh exposure to cold, when the same treatment was followed by an equally fortunate result. He then prescribed the remedy to several of his patients, all of whom were benefitted in the same way. He believes that this treatment is only suitable in acute cases.—*British Medical Record.*

To-day the British Museum, which receives every new book printed in Great Britian, has eight hundred thousand volumes upon its shelves. There are five other European libraries averaging over-half a million books. There are sixteen with over a quarter-million. The United States has accumulated; yet it is safe to calculate that a quarter of a million different books may be found in the libraries in New York City, and almost as many in Philadelphia, Boston and Washington. To read the mere titles of all the books in the Astor Library would occupy a rapid reader many days. Near five thousand new books appear in the English language every year.—H. M. Maccraken in the *National Literary Monthly.*

